# The Dual Role of Microglia in ALS: Mechanisms and Therapeutic Approaches

**DOI:** 10.3389/fnagi.2017.00242

**Published:** 2017-07-25

**Authors:** Maria Concetta Geloso, Valentina Corvino, Elisa Marchese, Alessia Serrano, Fabrizio Michetti, Nadia D’Ambrosi

**Affiliations:** ^1^Institute of Anatomy and Cell Biology, Università Cattolica del Sacro Cuore Rome, Italy; ^2^IRCCS San Raffaele Scientific Institute, Università Vita-Salute San Raffaele Milan, Italy; ^3^Department of Biology, University of Rome Tor Vergata Rome, Italy

**Keywords:** amyotrophic lateral sclerosis, M1/M2 microglia, neuroinflammation, anti-inflammatory drugs, genetic modifiers, mutant SOD1 mice

## Abstract

Amyotrophic lateral sclerosis (ALS) is a neurodegenerative disease characterized by a non-cell autonomous motor neuron loss. While it is generally believed that the disease onset takes place inside motor neurons, different cell types mediating neuroinflammatory processes are considered deeply involved in the progression of the disease. On these grounds, many treatments have been tested on ALS animals with the aim of inhibiting or reducing the pro-inflammatory action of microglia and astrocytes and counteract the progression of the disease. Unfortunately, these anti-inflammatory therapies have been only modestly successful. The non-univocal role played by microglia during stress and injuries might explain this failure. Indeed, it is now well recognized that, during ALS, microglia displays different phenotypes, from surveillant in early stages, to activated states, M1 and M2, characterized by the expression of respectively harmful and protective genes in later phases of the disease. Consistently, the inhibition of microglial function seems to be a valid strategy only if the different stages of microglia polarization are taken into account, interfering with the reactivity of microglia specifically targeting only the harmful pathways and/or potentiating the trophic ones. In this review article, we will analyze the features and timing of microglia activation in the light of M1/M2 phenotypes in the main mice models of ALS. Moreover, we will also revise the results obtained by different anti-inflammatory therapies aimed to unbalance the M1/M2 ratio, shifting it towards a protective outcome.

## ALS as a Composite Disease

Amyotrophic lateral sclerosis (ALS) is a multifactorial disease caused by genetic and non-inheritable components leading to motoneuron degeneration in the spinal cord, brain stem and primary motor cortex (Al-Chalabi and Hardiman, 2013). Most of ALS cases are sporadic (sALS), while 5%–20% report a familial history of the disease (fALS; Al-Chalabi et al., 2017). sALS and fALS share most neuropathological features and, from a clinical perspective, they appear very similar (Talbot, 2011). Pathological hallmarks characterizing degenerating motoneurons are cytoplasmic inclusions containing aggregated/ubiquitinated proteins as well as RNAs. Indeed, protein misfolding, with endoplasmic reticulum (ER) stress, impaired autophagy and damage to cytoskeleton are intracellular mechanisms involved in the pathogenesis of the disease (Taylor et al., 2016). However, ALS appears as a composite syndrome where the aberrant cellular pathways may not derive solely from a conformational issue, but involve many aspects of cellular physiology: RNA processing and mitochondria homeostasis are compromised, oxidative stress is increased, excitotoxic pathways are enhanced, neurotrophic support is reduced, glial inflammatory response is oriented towards an harmful side (Rossi et al., 2016). Actually, more than 40 genes have been found mutated in ALS, affecting numerous cellular functions (Al-Chalabi et al., 2017), the most relevant of which are: a hexanucleotide repeat (GGGGCC) expansion in an intron of the C9orf72 gene (Dejesus-Hernandez et al., 2011; Renton et al., 2011), supposed to generate toxic RNA species, loss of protein and/or harmful dipeptide-repeats formation (Haeusler et al., 2016); superoxide dismutase 1 (SOD1; Rosen et al., 1993), forming toxic aggregates and interfering with mitochondrial functions and autophagy (Turner and Talbot, 2008). In this regard, transgenic SOD1 mice are so far the most widely used model to study ALS. Both active (SOD1^G93A^, SOD1^G37R^) and inactive (SOD1^G85R^) mutants show a phenotype characterized by a progressive paralysis and death (at 5, 7 and 8.5 months, respectively), caused by degeneration of motoneurons (limited to 40% in SOD1^G85R^ mice), and exhibit gliosis within the spinal cord, brain stem and cortex (Philips and Rothstein, 2015), suggesting that neurodegeneration relies on a gain of toxic function of the protein. Other mutated proteins are fused in sarcoma (FUS; Kwiatkowski et al., 2009; Vance et al., 2009) and TAR-DNA binding protein-43 (TDP-43; Neumann et al., 2006), involved in the maturation of mRNAs, found in cytoplasmic inclusions (Guerrero et al., 2016); proteins regulating cytoskeleton architecture, such as profiling-1 (Wu et al., 2012; Yang et al., 2016), and vesicle trafficking, as vesicle-associated membrane protein/synaptobrevin-associated membrane protein B (Nishimura et al., 2004; Tsuda et al., 2008); autophagy-linked proteins, among which sequestosome 1 (Teyssou et al., 2013), optineurin (Nakazawa et al., 2016) and TANK-binding protein kinase-1 (TBK-1; Cirulli et al., 2015; Freischmidt et al., 2015). Mutations in these genes also affect the function of cell types other than motoneurons. Indeed, ALS is non-cell autonomous, as astrocytes and microglia can participate to determine the disease phenotype by a local inflammatory response (neuroinflammation) and characterized by phenotypic transition, migration to the site of injury, proliferation and secretion of pro-inflammatory mediators (Philips and Rothstein, 2014). Glial activation leads to changes in the expression of a wide range of genes related to the production of soluble molecules, such as cytokines and chemokines, damage–associated molecular patterns (DAMPs), reactive nitrogen and oxygen species (ROS), giving rise to profound modifications in their interactions with neurons (Becher et al., 2017). Actually, a noticeable level of neuroinflammation has been detected in both sALS and fALS, as well as in transgenic models of the disease (Troost et al., 1989; Engelhardt and Appel, 1990; Schiffer et al., 1996; Hall et al., 1998; Henkel et al., 2004, 2006). Signs of microglia reactivity have been detected well before overt symptoms onset (Brites and Vaz, 2014; Tang and Le, 2016), concomitantly with loss of neuromuscular junctions (Gerber et al., 2012) and early motoneuron degeneration (Alexianu et al., 2001).

The role of microglia has been strengthened by recent studies opening new perspectives in the knowledge of the non-cell autonomous molecular pathways possibly contributing to ALS.

Lack of C9orf72 in a loss-of-function model of the disease produced no signs of motoneuron degeneration, but led to lysosomal accumulation and altered immune responses in macrophages and microglia (O’Rourke et al., 2016). Furthermore, the recently described ALS-susceptibility gene, TBK1, not only has a central function in autophagy processes, but is involved in innate immunity signaling pathways, regulating the production of interferon α (IFN α) and IFN β (Ahmad et al., 2016). A close relation between disruption of the autophagy machinery and microglial activation has been recently proposed (Plaza-Zabala et al., 2017): hence, an impaired autophagy linked to modifications in the response to pro-inflammatory stimuli and pathogen clearance by resident immune cells likely contributes to the etiopathology of the disease. Recent data show an earlier and more detrimental clinical course in SOD1^G93A^ mice lacking telomerase (Linkus et al., 2016), evidencing therefore a possible aging effect on microglia priming in ALS. Indeed aged and mutant SOD1 (mSOD1)-expressing microglia display a common signature of gene expression, as well as specific patterns (Holtman et al., 2015).

In this review article, we therefore describe how the adaptive phenotypes of microglia participate to neurodegeneration in ALS, evidencing how the concept of a bipolar, protective vs. harmful, response of microglia has been rapidly changed in less than a decade. We also discuss how anti-inflammatory drugs have been used to polarize microglia towards a neuroprotective signature to control the extent of activation and if and how this has reached therapeutic benefits.

## M1/M2 Phenotype in ALS

### Overview

Microglia are largely considered as the brain’s resident immune cell, which has been classically described to exist in two states, resting and activated (Cherry et al., 2014). In the adult healthy brain, two-photon imaging showed that the so called “resting” microglia is, in actual facts, a highly dynamic population (Nimmerjahn et al., 2005), which actively screen their microenvironment with motile processes, exerting a crucial role in maintaining homeostasis (Luo and Chen, 2012). It is indicated as “surveillant” microglia and participates to many physiological functions, including synaptic pruning, adult neurogenesis and modulation of neuronal networks (Walton et al., 2006; Kettenmann et al., 2013).

This highly specific interaction with the extracellular environment is tightly regulated (Nimmerjahn et al., 2005; Parisi et al., 2016b), therefore these cells rapidly react to abnormalities, adopting a less ramified/amoeboid phenotype, corresponding to activated microglia (Luo and Chen, 2012; Cherry et al., 2014). Similarly to peripheral macrophages, the term activation has been associated at least with two distinct phenotypes, M1 (toxic) and M2 (protective), in response to different microenvironmental signals, in turn involved in the production of a variety of effector molecules (Du et al., 2016). Microglia recognize pathogens via pattern recognition receptors, which interact with classes of DAMPs derived from exogenous microorganisms or endogenous cell types involved in immunity processes, respectively. The interaction triggers a downstream gene induction program aimed at initiating cellular defense mechanisms, including the release of inflammatory cytokines and chemokines (Colton, 2009; Kigerl et al., 2014).

In particular, in *in vitro* settings, lipopolysaccharide (LPS) or IFN-γ stimulate “classically activated” M1 microglia, which release pro-inflammatory mediators. They include pro-inflammatory cytokines (interleukin [IL]-1α, IL-1β, IL-6, IL-12, IL-23, tumor necrosis factor-α [TNF-α]), chemokines, prostaglandin E2, chemokine (C-C motif) ligand 2, ROS and inducible nitric oxide synthase (iNOS; Bagasra et al., 1995; Du et al., 2016; Orihuela et al., 2016).

In contrast, “alternatively activated” M2 phenotype, which is induced by anti-inflammatory cytokines IL-4, IL-10 or IL-13, suppresses inflammation, clears cellular debris through phagocytosis, promotes extracellular matrix reconstruction and supports neuron survival through the release of protective/trophic factors (Hu et al., 2015; Du et al., 2016; Tang and Le, 2016). “Acquired deactivation” represents another M2 anti-inflammatory phenotype and it is mainly induced by the uptake of apoptotic cells or exposure to anti-inflammatory cytokines, such as IL-10 and transforming growth factor-β (Tang and Le, 2016).

### Microglia in ALS

**Figure 1:**
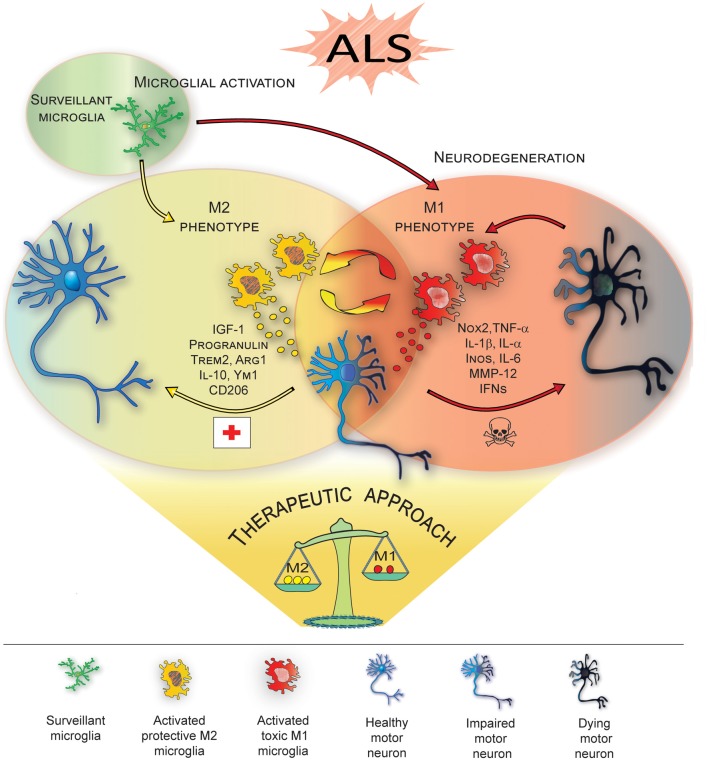
M1/M2 microglia polarization during amyotrophic lateral sclerosis (ALS)-induced motor neuron degeneration. During ALS progression activated microglia represent a continuum between the neuroprotective M2 phenotype, which promotes tissue repair and supports neuron survival by releasing neuroprotective factors, vs. the toxic M1, which produces cytokines increasing inflammation and further supporting M1 polarization, thus contributing to neuronal death. Therapeutic approaches targeting microglia polarization and resulting in induction of the M2 phenotype are promising strategies to ameliorate local neurodegeneration and improve the clinical outcome of the disease (see Table 1 for details).

Studies investigating the progression of the disease in ALS mice indicate that, *in vivo*, resident microglia increase their number during disease progression, and their activation states represent a continuum between the two classical phenotypes, i.e., neuroprotective M2 vs. toxic M1 (Liao et al., 2012; Chiu et al., 2013; Figure 1). In line with this, the occurrence of two different phenotypes of microglial cells, on the basis of their morphology, has been recently described in SOD1^G93A^ transgenic mice: type “R1”, showing short and poorly branched processes, which represents the vast majority of microglia in the early-stage of the disease and corresponding to early transformation of surveillant microglia, and type “R3” microglia, exhibiting large cell bodies with short and thick processes, which are typical of end-stage phases of the disease (Ohgomori et al., 2016). Consistently, microglia have been shown to exhibit, at the pre-onset phase of SOD1-mediated disease, an anti-inflammatory profile with attenuated TLR2 responses to controlled immune challenge, and a overexpression of anti-inflammatory IL-10 (Gravel et al., 2016). Subsequently, at disease onset and during the slowly progressing phase, the prevalent expression of specific M2 markers, (e.g., Ym1 and CD206), was detected in the lumbar spinal cords of ALS mice (Beers et al., 2011a). Eventually, in end-stage animals, a microglial phenotype expressing high levels of NOX2, the subunit of nicotinamide-adenine-dinucleotide-phosphate oxidase expressed by macrophages considered M1 prototypic marker, appears to be prevalent (Beers et al., 2011b).

M1 ALS microglia appear hyper-reactive to inflammatory stimuli (D’Ambrosi et al., 2009) and the specific role of mutated proteins in driving this increased toxicity has been suggested by many studies (Beers et al., 2006; Xiao et al., 2007; Liao et al., 2012). Mutant forms of TDP-43 are able to activate microglia and upregulate the release of pro-inflammatory mediators, including NOX2, TNF-α and IL-1β (Zhao et al., 2015). Consistently, also the intracellular expression of high levels of TDP-43 underlies the occurrence of a more toxic microglial phenotype, when stimulated, *in vitro*, with LPS or ROS (Swarup et al., 2011).

Similarly, exogenous SOD1^G93A^ or SOD1^G85R^ induce, *in vitro*, morphological and functional activation of microglia, increasing their release of pro-inflammatory cytokines and ROS (Zhao et al., 2010). In chimeric mice with both normal and mSOD1-expressing cells, non-neuronal cells that do not express mSOD1, including microglia, delay degeneration and significantly extend survival of mutant protein-expressing motoneurons (Clement et al., 2003). Interestingly, also mSOD1-expressing microglia underlie phenotypic transformation during the disease. More specifically, evidence has been provided that, when co-culturing different-aged mSOD1 microglia with WT motoneurons, mSOD1-expressing early-activated microglia exhibit neuroprotective features, enhancing neuronal survival, while end-stage derived mSOD1 microglia show toxic properties, increasing neuronal death rate (Liao et al., 2012). Additionally, mSOD1 microglia shows increased expression of molecular players of the ER stress pathway (Ito et al., 2009), which may be involved in their toxic phenotype.

At the molecular level, mutated proteins, including TDP-43 and FUS, induce the selective activation of nuclear factor-kappa B (NF-kB), master regulator of inflammation (Frakes et al., 2014).

On this basis, the possibility to appropriately modulate microglial phenotypes, enhancing the anti-inflammatory properties and inhibiting or reducing M1 toxicity, could be a promising therapeutic strategy for ALS, therefore a comprehensive knowledge of both timing and molecular players of microglial activity is needed. However, emerging evidence suggests that the M1/M2 paradigm seems to be an oversimplification (Ransohoff, 2016) and substantial differences between microglia and peripheral macrophages, from which the terminology derives, should be carefully considered. As resident macrophages of the brain, microglia have an elaborate repertoire of brain specific functions, sustained by a peculiar gene expression profiling (Gautier et al., 2012). *In vitro*, phenotypic redirection is a feature of peripheral macrophages, while microglia exhibit a lower grade of plasticity (Parisi et al., 2016b). Coexistence of the two opposite phenotypes, more than transition from M2 to M1, during ALS progression has also been recently highlighted by several findings. For instance, beneficial components of inflammation, such as insulin growth factor-1 (IGF-1), whose release is suppressed in a pro-inflammatory (M1) environment but encouraged in an M2 protective environment (Suh et al., 2013), is overexpressed by SOD1^G93A^ microglia not only in pre-symptomatic stage, but also in end-stage (Chiu et al., 2008). Furthermore, a down-regulation of IL-6 over time, associated with an up-regulation of IL-1R antagonist, has been reported, suggesting the occurrence of an anti-inflammatory response (Chiu et al., 2008). Analysis of transcriptome changes of SOD1^G93A^ microglia essentially confirmed these observations. They also evidenced that the activation of genes involved in anti-inflammatory pathways, including, *Igf1*, *Progranulin* and *Trem2*, coexists with the upregulation of genes related to potentially neurotoxic factors, among which Matrix metalloproteinase-12 and classical proinflammatory cytokines, (Chiu et al., 2013). Interestingly, critical differences in gene expression profiling among M1/M2 macrophages, LPS-activated microglia and SOD1^G93A^ activated microglia emerge: while LPS-activated microglia show enriched in DNA replication-, cell cycle- and innate immune signaling-genes, SOD1^G93A^ activated microglia are enriched in the transcripts of genes related to neurodegenerative diseases, e.g., AD, Huntington’s and Parkinson’s disease, suggesting a neurodegeneration-specific signature for ALS microglia. More interestingly, SOD1^G93A^-expressing microglia do not display a significant prevalence of M1 or M2 phenotypes at any time point during disease progression (Chiu et al., 2013). In line with this results, an increased expression of both iNOS (M1 marker) and arginase 1 (Arg1; M2 marker) has been shown to parallel the generalized increase of activated microglia in SOD1^G93A^ mice (Lewis et al., 2014). Consistently, characteristics different from typical M1 or M2 phenotypes have been reported in end-stage SOD1^G93A^ rats, which also show predominant microglial activation in most severely affected regions (lumbar spinal cord), as if several phenotypically different microglial subpopulations were present throughout differently affected regions of the CNS (Nikodemova et al., 2014).

## Microglial Switch and Therapeutic Approaches in ALS Animal Models

Targeting the microglia has been the focus of neuroprotective strategies, based on pharmacological or genetic approaches, aimed at modulating microglia reactivity in the attempt to improve the clinical outcome in animal models of the disease (Table 1, Figure 1). In this regard, pioneer studies based on administration of minocycline, a tetracycline antibiotic that prevents microglial activation, showed that, when administered in both SOD1^G93A^ and SOD1^G37R^ mice before disease onset, it attenuates microglial activation and delays disease onset and mortality (Kriz et al., 2002; Van Den Bosch et al., 2002; Zhu et al., 2002). On the other hand, when administered after the onset of the disease, it fails to improve clinical and/or pathological features, even increasing microgliosis (Keller et al., 2011). Interestingly, recent findings obtained in SOD1^G37R^ mice have shown that minocycline specifically attenuates the M1 phenotype, without influencing the expression of M2 markers (Kobayashi et al., 2013; Table 1), thus highlighting the crucial role exerted by the modulation of M1/M2 balance in the therapeutic effectiveness.

**Table 1:** Preclinical approaches affecting microglia M1/M2 phenotype in transgenic mutant superoxide dismutase1 (mSOD1) mice.

Drug administered/Genes silenced	Action/Function	M1 modulation	M2 modulation	Outcomes
AMD3100 (Rabinovich-Nikitin et al., 2016)	CXCR4 antagonist	↓TNF-α, IL-6		Survival +10%, ↑onset, b.w., motor function
BBG (Apolloni et al., 2014)	P2X7 antagonist	↓NOX2, IL-1β; n.s.c. TNF-α, IL-6, iNOS	↑BDNF, IL-10	Survival n.s.c., ↑motor function
Bee venom (Yang et al., 2010)	anti-inflammation	TNF-α↓		Survival +18%, ↑onset, motor function
Celastrol (Kiaei et al., 2005b)		iNOS↓		Survival +13%, ↑onset, b.w., motor function
Celecoxib/Rofecoxib + Creatine (Klivenyi et al., 2004)	COX-2 inhibitor	PGE2↓		Survival +30%, ↑b.w., motor function
Clemastine (Apolloni et al., 2016b)	Antihistamine	↓CD68, gp91^phox^	↑Arg1, BDNF	Survival n.c.s., ↑onset,
DL-NBP (Feng et al., 2012)	Neuroprotection	↓TNF-α		Survival +42%, ↑b.w., motor function
EGCG (Xu et al., 2006)	Neuroprotection	iNOS↓		Survival +10%, onset +9%,
hMSC (Zhou et al., 2013)	Stromal cells	↓TNF-α, iNOS		Survival +10%, onset +6%, ↑motor function
IL-1RA (Meissner et al., 2010)	IL-1R antagonist			Survival +4%, ↑motor function
Lenalidomide (Kiaei et al., 2006)	↓ TNF-α	↓TNF-α, IL-1α, IL-1β	↑TGF-β1	Survival +18%, ↑onset, b.w., motor function
*M-CSF (Gowing et al., 2009)	Cytokine	↑TNF-α, IL-1β;↓IL-6, NOX2	↓IL-4; ↑TGF-β1	Survival −3%
Minocycline (Kobayashi et al., 2013)	↓glia activation	↓TNF-α, IL-1β, INF-γ, CD86, CD68	n.s.c. CD206, Arg1, IL-4, IL-10, Ym1	Survival +54%, onset +15%
Nimesulide (Pompl et al., 2003)	COX-2 inhibitor	PGE2↓		Survival n.d., ↑onset, motor function
Pioglitazone (Kiaei et al., 2005a)	PPARγ agonist	↓iNOS, COX2		Survival +13%, ↑onset, b.w., motor function
R723 (Tada et al., 2014)	JAK2 inhibitor	↓CD11b, iNOS; n.s.c. TNF-α, IL6, IL-1β	n.s.c. Arg1, Ym1, IL-4	n.s.c.
scAAV9-VEGF (Wang et al., 2016)	↑ VEGF	↓TNF-α, CD68	↑Arg1, Ym1	Survival +10%, ↑b.w., motor function
Sulindac (Kiaei et al., 2005c)	COX inhibitor	COX2↓		Survival +10%, ↑b.w., motor function
Thalidomide (Kiaei et al., 2006)	↓ TNF-α	↓TNF-α; n.s.c. IL-1α, IL-1β	↑TGF-β1	Survival +12%, ↑onset, b.w., motor function
gp91^phox^^−^ (Wu et al., 2006)	NOX2 inhibition	IL-1β n.s.c.		Survival +11%
IL-1β^−/−^ (Meissner et al., 2010)	IL-1β decrease	↓IL-1β^−/−^		Survival +5%
iNOS^−/−^ (Martin et al., 2007)	iNOS inhibition	↓iNOS		↑Survival
NOX2^−/−^ (Marden et al., 2007)	NOX2 inhibition	↓NOX2		Survival +73%, ↑onset, b.w., motor function
**TNF-α^−/−^ (Gowing et al., 2006)	TNF-α decrease	↓TNF-α		n.s.c.
*xCT^−/−^ (Mesci et al., 2015)	↓ Glutamate release	Onset: ↑IL-1β, iNOS E.s.: ↓IL-1β, iNOS	Onset: ↓Arg1, Ym1 L.s.:↑Arg1, Ym1	Survival n.s.c., onset −9%, ↑b.w. (at l.s.), motor function

All trials were performed in SOD1^G93A^ mice except the cases indicated with asterisks (* performed in SOD^G37R^ mice, ** performed both in SOD^G93A^ and SOD^G37R^ mice). The reported data refer to most effective results obtained in the cited article. Abbreviations: n.d., not described, n.s.c., non significant changes, ↑ increased, ↓ decreased; b.w., body weight, e.s., end stage, l.s., late symptomatic stage.

Hence, pharmacological modulation of molecular pathways related to microglial polarization has been explored. The hyperactivation of P2X7 receptors, strongly involved in neuroinflammatory response (Burnstock, 2008; Apolloni et al., 2009; Volonté et al., 2012; Sperlágh and Illes, 2014), has been described in microglia of both ALS patients and animal models (Yiangou et al., 2006; D’Ambrosi et al., 2009), where it is associated to the production of pro-inflammatory factors, including miR-125b (D’Ambrosi et al., 2009; Parisi et al., 2013, 2016a). Consistently, the administration of the P2X7 antagonist Brilliant Blue G (BBG), within a critical time frame, improves several features of the disease (Cervetto et al., 2013; Apolloni et al., 2014). BBG neuroprotection, obtained at late pre-onset administration, is supported by the upregulation of IL-10 and BDNF, associated to M2 phenotype, together with a reduction of NF-kB protein, NOX-2 and IL1β, markers of M1 polarization (Table 1). However, BBG administration at earlier phases fails to counteract disease progression. In this case, although it reduces M1 markers, it does not affect the expression of M2 mediators, whose neuroprotective properties seem to be essential to improve the clinical outcome (Apolloni et al., 2014).

Microglia-mediated neuroinflammation is also modulated by histamine (Ferreira et al., 2012; Volonté et al., 2015; Barata-Antunes et al., 2017). The antihistamine drug Clemastine, administered to SOD1^G93A^ mice at the asymptomatic phase until the end-stage of disease, fails to improve clinical symptoms and lifespan, although it modulates the M1/M2 balance by reducing CD68, NOX2 and P2X7 expression and concomitantly up-regulating Arg1 (Apolloni et al., 2016b; Table 1). Conversely, when administered at the asymptomatic phase to the onset, it delays the disease onset and improves the motor functions and survival rate (Apolloni et al., 2016a). Clemastine also activates autophagy in SOD1^G93A^ primary microglia, thus suggesting that targeting autophagy in microglia could be a promising therapeutic strategy (Apolloni et al., 2016a).

Alternative therapeutic strategies to shift the balance towards the M2 phenotype involve the use of trophic factors. Several findings showed that the delivery of viral vectors encoding growth factors, such as IGF-1, glial-derived neurotrophic factor, vascular endothelial growth factor (VEGF) extends lifespan and slows the progression of the disease in ALS animal models (Acsadi et al., 2002; Kaspar et al., 2003; Azzouz et al., 2004; Dodge et al., 2010; Wang et al., 2016). Interestingly, the intrathecal injection of self-complementary adeno-associated-virus (scAAV)9-VEGF at disease onset decreases TNF-α, IL-1β and CD68 levels and increases those of Arg-1 and Ym-1 (Table 1), showing that the modulation of M1/M2 balance could support the protective effects correlated to VEGF administration (Wang et al., 2016).

Further, the deletion of the cystine/glutamate-antiporter xCT/Slc7a11 (xCT), a critical glial transporter system involved in the excessive glutamate release from M1 microglia, has provided additional finding on this matter. Indeed, xCT deletion at the early-stages of the disease, in fact, increases the expression of M1 marker IL1β and concurrently reduces M2 marker Ym1/Chil3, thus resulting in earlier disease onset. Conversely, lack of xCT, at the end-stage, increases Ym1/Chil3 and Arg1 expression, which possibly sustains the delay of disease progression (Mesci et al., 2015; Table 1).

These data underline that the modulation of microglia-specific pathways may ameliorate local neurodegeneration. However, growing evidence suggests that a successful therapeutic strategy for ALS could be obtained only interfering with different pathways in different cell types. In light of this, it was recently demonstrated that microglial NF-κB suppression combined with mSOD1 reduction in astrocytes and motoneurons results not only in attenuated neuroinflammation and neurodegeneration, but also increases mice mean survival (Frakes et al., 2017), demonstrating that the redirection of microglia polarization may still be an effective strategy to counteract ALS when associated with the interception of other pathogenic mechanisms.

## Author Contributions

MCG and ND wrote respectively section 2 and 1 and conceived, designed and revised the manuscript; VC wrote section 3; EM prepared the artwork; AS created the table; FM revised the work.

## Conflict of Interest Statement

The authors declare that the research was conducted in the absence of any commercial or financial relationships that could be construed as a potential conflict of interest.
